# Aminorex, a metabolite of the cocaine adulterant levamisole, exerts amphetamine like actions at monoamine transporters^☆^

**DOI:** 10.1016/j.neuint.2013.11.010

**Published:** 2014-07

**Authors:** Tina Hofmaier, Anton Luf, Amir Seddik, Thomas Stockner, Marion Holy, Michael Freissmuth, Gerhard F. Ecker, Rainer Schmid, Harald H. Sitte, Oliver Kudlacek

**Affiliations:** a Institute of Pharmacology, Center for Physiology and Pharmacology, Medical University of Vienna, Waehringerstrasse 13A, 1090 Vienna, Austria; b Clinical Department of Laboratory Medicine, Medical University of Vienna, Waehringer Guertel 10-20, 1090 Vienna, Austria; c University of Vienna, Department of Medicinal Chemistry, Althanstrasse 14, 1090 Vienna, Austria

**Keywords:** SERT, serotonin transporter, NET, norepinephrine transporter, DAT, dopamine transporter, 5-HT, serotonin, DA, dopamine, KHB, Krebs–Ringer–HEPES buffer, HPLC, high performance liquid chromatography, LC–MS, liquid chromatography–mass spectrometry, Levamisole, Aminorex, Neurotransmitter transporter, Cocaine, Adulterant

## Abstract

Psychostimulants such as amphetamine and cocaine are illicitly used drugs that act on neurotransmitter transporters for dopamine, serotonin or norepinephrine. These drugs can by themselves already cause severe neurotoxicity. However, an additional health threat arises from adulterant substances which are added to the illicit compound without declaration. One of the most frequently added adulterants in street drugs sold as cocaine is the anthelmintic drug levamisole. We tested the effects of levamisole on neurotransmitter transporters heterologously expressed in HEK293 cells. Levamisole was 100 and 300-fold less potent than cocaine in blocking norepinephrine and dopamine uptake, and had only very low affinity for the serotonin transporter. In addition, levamisole did not trigger any appreciable substrate efflux. Because levamisole and cocaine are frequently co-administered, we searched for possible allosteric effects; at 30 μM, a concentration at which levamisole displayed already mild effects on norepinephrine transport it did not enhance the inhibitory action of cocaine. Levamisole is metabolized to aminorex, a formerly marketed anorectic drug, which is classified as an amphetamine-like substance. We examined the uptake-inhibitory and efflux-eliciting properties of aminorex and found it to exert strong effects on all three neurotransmitter transporters in a manner similar to amphetamine. We therefore conclude that while the adulterant levamisole itself has only moderate effects on neurotransmitter transporters, its metabolite aminorex may exert distinct psychostimulant effects by itself. Given that the half-time of levamisole and aminorex exceeds that of cocaine, it may be safe to conclude that after the cocaine effect “fades out” the levamisole/aminorex effect “kicks in”.

## Highlights

• We quantified adulterants in street drugs sold as cocaine.
• We analyzed effects of the most common adulterant levamisole, on neurotransmitter transporters.
• Differences in the selectivity of levamisole can be explained by homology modelling and docking.
• Aminorex, a metabolite of levamisole, modulates neurotransmitter transporters directly.
• Depending on the transporter, aminorex acts as a blocker or as a releaser.

## 1 Introduction

Monoamine transporters for serotonin (SERT), norepinephrine (NET) and dopamine (DAT) belong to the family of Na^+^/Cl^−^-dependent neurotransmitter transporters and remove their substrates to end synaptic transmission (Kristensen et al., 2011). Apart from this physiological role, these transporters are the targets of illicit drugs like cocaine or amphetamines (Rothman and Baumann, 2003). Amphetamines lead to a reverse action of all of these transporters and to a number of other intracellular effects which actively increase the concentration of neurotransmitters in the synaptic cleft (Sitte and Freissmuth, 2010). In contrast, cocaine raises the synaptic concentration of monoamines by inhibiting the activity of these transporters. Both classes of compounds are sold on the street market for illicit drugs at the risk of the users because both the quality and identity of the purchased drugs are without any control. This situation is alleviated by the government-supported Viennese drug prevention project ‘checkit! Check your drugs’, which offers cost-free and anonymous analyses of drugs. Thereby drug consumers gain information about the contents of their drug as well as possible risks of those compounds. Importantly and often to the great surprise of the user, the purchased drug does not contain the compound under the name it was sold. Recently, a survey of unknown street drugs from the ‘checkit!’ project revealed that a combination of amphetamine and *m*-chlorophenylpiperazine (*m*CPP) was sold under the name of methylene-dioxymethamphetamine (MDMA, ‘ecstasy’; (Rosenauer et al., 2013)). Hence, the combinations of two active drugs are common (Schifano et al., 2011). However, drugs are also adulterated with more or less psychoactive active compounds: amphetamines are often mixed with e.g. caffeine (Vanattou-Saifoudine et al., 2012) and cocaine has been found to be mixed with a wide variety of adulterants. One prominent example of these adulterants is levamisole (Fig.1A) which has been found in most of the drug samples sold as cocaine in the past. Levamisole is used by veterinarians as an anthelmintic drug (Martin et al., 2012); its mode of action is the stimulation of ionotropic acetylcholine receptors (AChR) resulting in calcium influx causing paralysis of the worms (Levandoski et al., 2003; Rayes et al., 2004). Under the trade name Ergamisol, levamisole was also used to treat worm infections in humans but had to be withdrawn from the U.S market in 2000 because of its severe side-effects (Renoux, 1980). Most recently, several drug consumers suffered from agranulocytosis after repeated intake of cocaine adulterated (“cut”) with levamisole (Muirhead and Eide, 2011; Wolford et al., 2012).

Several plausible explanations exist why levamisole is used as a cocaine-adulterant: (i) levamisole was reported to improve the mood of patients and induced insomnia and hyperalertness (Mutch and Hutson, 1991). (ii) The chemical properties of levamisole are similar to cocaine; for instance, color and melting point render both drugs almost indistinguishable without further chemical analysis (Chang et al., 2010). (iii) The use of levamisole as a drug in veterinary medicine makes it easily available and keeps the costs low (Waller, 2006). (iv) Levamisole was found to be rapidly metabolized in the human body to aminorex and related metabolites (Hess et al., 2013; Reid et al., 1998). Aminorex (Fig.1A) is an amphetamine-like agent that was detected in racehorses after levamisole administration (Barker, 2009). Moreover, aminorex was detected in human urine samples in a multitude of cocaine abusers (Bertol et al., 2011; Karch et al., 2012). Aminorex was marketed as an appetite suppressant in the mid-1960s mainly in Switzerland, Austria, and Germany; it was found to cause pronounced vasoconstriction in the pulmonary vasculature (Byrne-Quinn and Grover, 1972; Stuhlinger et al., 1969; Rothman et al., 1999) and was withdrawn in 1972 due to several cases of fatal and life-threatening pulmonary hypertension (Fishman, 1999a).

In the present work, we examined whether levamisole exerts direct effects on neurotransmitter transporters and compared these to the action of its metabolite, aminorex.

## 2 Materials and methods

Dulbecco’s modified Eagle’s medium (DMEM) and trypsin were purchased from PAA Laboratories GmbH (Pasching, Austria). Fetal calf serum was purchased from Invitrogen. [^3^H]5-HT ([^3^H]5-hydroxytryptamine; [^3^H]serotonin; 28.3 μCi/mmol) and [^3^H]DA ([^3^H]dihydroxyphenylethylamine, [^3^H]dopamine; 46 μCi/mmol) were purchased from PerkinElmer, Boston, MA. [^3^H]1-Methyl-4-phenylpyridinium ([^3^H]MPP^+^; 85 μCi/mmol) was supplied by American Radiolabeled Chemicals (St. Louis, MO). Paroxetine was from Santa Cruz Biotechnology, mazindole, serotonin, levamisole, cocaine, aminorex, nisoxetine, D-amphetamine, and monensin were purchased from Sigma–Aldrich Co.

### 2.1 Sample collection and analysis

The samples used in this study were obtained from drug users participating voluntarily and anonymously in the ‘checkit!’ drug prevention program. Three to ten milligrams of substance were scraped into a tapered 2 ml test vial and weighed with an analytical balance. The substance was dissolved in 1 mL of methanol and vortex mixed for 1 min. The solution was centrifuged for 3 min at 10,000*g* in an Eppendorf centrifuge. Ten microliters of the supernatant were diluted with 0.4 mL of internal standard solution (trazodone 50 μg/mL dissolved in 10 mM aqueous ammonium formate buffer), 2 μl of the solution was analysed with reversed phase HPLC and LC/mass spectrometry coupling as described in a previous study (Rosenauer et al. 2013).

### 2.2 Uptake and release assays

The generation of HEK293 cell lines expressing the human isoforms of SERT, NET, or DAT (HEK-SERT, HEK-DAT, or HEK-NET, respectively) was described earlier (Scholze et al., 2002). HEK293 cells stably expressing either neurotransmitter transporter were seeded onto poly-*d*-lysine-coated 96-well plates (40,000 cells/well), 24 h prior to the experiment. For inhibition experiments, the specific activity of the tritiated substrate was kept constant: [^3^H]DA, 0.1 μM; [^3^H]MPP+, 0.015 μM; [^3^H]5-HT, 0.1 μM. Assay conditions were used as outlined earlier (Sucic et al., 2010). In brief, the cells were washed twice with Krebs–Ringer–HEPES buffer (KHB; composition: 25 mM HEPES·NaOH, pH 7.4, 120 mM NaCl, 5 mM KCl, 1.2 mM CaCl_2_, and 1.2 mM MgSO_4_ supplemented with 5 mM d-glucose). Then, the diluted reference and sample compounds were added and incubated for 5 min to allow for equilibration with the transporters. Subsequently, the tritiated substrates were added and the reaction was stopped after 1 min (SERT and DAT) and 3 min (NET), respectively. Cells were lysed with 1% SDS and the released radioactivity was quantified by liquid scintillation counting. All determinations were performed in duplicate or triplicate.

For release studies, HEK-SERT, HEK-NET, or HEK-DAT cells were grown overnight on round glass coverslips (5-mm diameter, 40,000 cells per coverslip) placed in a 96-well plate and preloaded with 0.4 μM [^3^H]dopamine, 0.1 μM [^3^H]MPP+, or 0.4 μM [^3^H]5-HT for 20 min at 37 °C in a final volume of 0.1 mL/well. Coverslips were then transferred to small superfusion chambers (0.2 ml) and superfused with KHB (25 °C, 0.7 ml × min^−1^) as described (Scholze et al., 2002). A washout period of 40 min established a stable baseline for efflux of radioactivity; thereafter, substances were added and the experiment was started with the collection of fractions (2 min). At the end of the experiment, cells were lysed in 1% SDS and the released radioactivity was quantified by liquid scintillation counting.

The release of [^3^H] labelled substrate was expressed as fractional rate (i.e., the radioactivity released within one fraction was expressed as a percentage of the total radioactivity present in the cells at the beginning of that fraction). Drug-induced release was calculated by subtracting the estimated basal release from total release during the first 8 min of drug exposure and is expressed as a percentage of radioactivity in the cell at the beginning of drug exposure. Data were normalized by using cpm values with no substance present (only solvent) as 100%. IC_50_ values were calculated using non-linear regression fits performed with Prism software (GraphPad 5.0, San Diego, CA, U.S.A.). Data transformed into Dixon plots were fitted by linear regression.

### 2.3 Homology modelling and docking

Levamisole has a *pK*_a_ value of 7. Both the neutral and protonated levamisole structures were built and minimized with QSite (version 5.8, Schrödinger, LLC) using the B3LYP method applying the 6-31G^∗^ basis set (Murphy et al., 2000). SERT and NET share over 90% sequence similarity with DAT. Homology models of human SERT and NET were generated with Modeller 9.12 (Sali and Blundell, 1993) using the validated human DAT model in the outward facing conformation (Stockner et al., 2013) as template. The best model out of the 250 generated was used for further studies. The models of SERT, DAT and NET were energy minimized with Molecular Operating Environment (MOE, 2012) applying the CHARMM22 forcefield (Brooks et al., 2009) and using position restrains of 100 kcal/mol on the backbone.

The induced fit docking protocol of the Schrödinger package was used for ligand docking into the central binding site (Glide version 5.8, Schrödinger, LLC, New York) using standard parameter setting (Sherman et al., 2005). The neutral and the protonated form of levamisole were docked as fully flexible molecules. The protonatable nitrogen of levamisole was constrained to interact with the central aspartate in the binding side, because the positive amine functional group of the endogenous substrates of SERT, DAT and NET has been shown to interact with the respective residue. Conformations of amino acid side chains within 6 Å distance to the ligand were optimized in the OPLS-AA 2005 force field after docking. Default energy levels were employed for selection and filtering of the poses.

The p*K*_a_ value of aminorex is 7.4. Both, neutral and protonated form of aminorex were docked using the same methods as for above levamisole.

## 3 Results and discussion

In 2012, 104 drug samples were obtained from drug users participating voluntarily and anonymously in the ‘checkit!’ program which were originally purchased as “cocaine”. We included all samples in our study and analyzed them by LC–MS. Two samples contained pure cocaine whereas seven samples were completely devoid of cocaine. The remaining 95 samples contained cocaine in varying amounts (Table 1). Importantly, LC–MS revealed a number of different adulterants that were mixed to cocaine: Fig. 1B shows a representative chromatogram. Among others we found paracetamol, benzoylecgonine, levamisole and phenacetin (Table 1); levamisole was present in almost two thirds of all examined samples (66 of 104 samples). The ratio between cocaine and levamisole in these samples was highly variable. While some samples contained less than 1% levamisole, one sample even displayed 20 times more levamisole than cocaine. The mean amount of levamisole was 59 ± 22% relative to cocaine. This highly variable amount of the different drugs also emphasize the risk incurred: people consume the purchased drug until they experience the desired effect (Cole et al., 2010). Hence, they are likely to also consume more of the adulterant.

### 3.1 Levamisole inhibits NET, DAT and SERT only at high concentrations

**Fig. 2:**
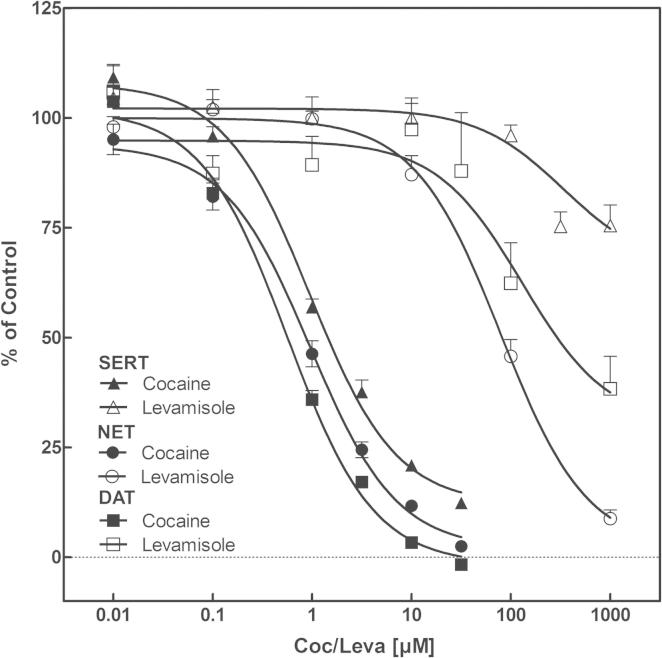
Uptake inhibition experiments. Uptake inhibitions with levamisole and cocaine were performed using HEK 293 cells stably expressing human DAT, NET or SERT. Therefore cells were incubated with tritiated compounds after a prior incubation for 5 min with test compound. Uptake of substrate (SERT: 5-HT; NET: MPP+; DAT: dopamine) at increasing levamisole, respectively cocaine concentrations is expressed as percentage of the maximum uptake without any inhibitory substance. IC_50_ values for levamisole (SERT: 1512 ± 1.09 μM; NET: 74.53 ± 1.12 μM; DAT: 209.9 ± 1.31 μM) (SERT: 1.8 ± 1.12 μM; NET: 1.1 ± 1.07 μM; DAT: 0.56 ± 1.12 μM). Data are mean ± SEM of four independent experiments.

Given the fact that in our survey levamisole was the most commonly used adulterant of cocaine, we reasoned that it likely has pharmacological properties that render it especially useful as adulterant. This conjecture is justified, because our findings are in line with other reports: levamisole has been observed to be one of the most predominant adulterants over the past two decades (Buchanan et al., 2010; Chai et al., 2011). Hence, we first explored whether levamisole exerted an action on the three main neurotransmitter transporters SERT, NET and DAT using HEK293 cells stably expressing the individual human isoforms of these transporters. Uptake-inhibition experiments were performed with increasing concentrations of levamisole or cocaine (Fig. 2). Cocaine blocked the uptake at the expected concentrations (Ravna et al., 2003): the observed IC_50_ values were 1.8 ± 1.12 μM (SERT), 1.0 ± 1.07 μM (NET) and 0.56 ± 1.12 μM (DAT). Levamisole also reduced the uptake of substrate but at much higher concentrations. Measured IC_50_ values were 1512 ± 1.09 μM (SERT), 74.5 ± 1.12 µM (NET), 209.9 ± 1.31 μM (DAT). Based on the high IC_50_ values of levamisole, it is unlikely that the compound exerts any significant inhibitory action on the transporters *in vivo*, when administered in therapeutic doses (e.g., as an adjuvant in cancer chemotherapy). Oral administration of 50 mg levamisole gives rise to peak plasma concentrations (*c*_max_) of on average 368 μg/L (equivalent to about 1.5 μM) (Gwilt et al., 2000). There is a large intraindividual variation in pharmacokinetics (Gwilt et al., 2000) and some uncertainty about nasal absorption. In addition, levamisole is a highly lipophilic substance that readily permeates the blood–brain barrier (Lin and Tsai, 2006). Therefore levamisole may possibly reach higher concentrations than cocaine in the brain and thereby lead to or support a blockage of NET and DAT, when consumed at excessive levels.

**Supplementary Fig. 3.1:**
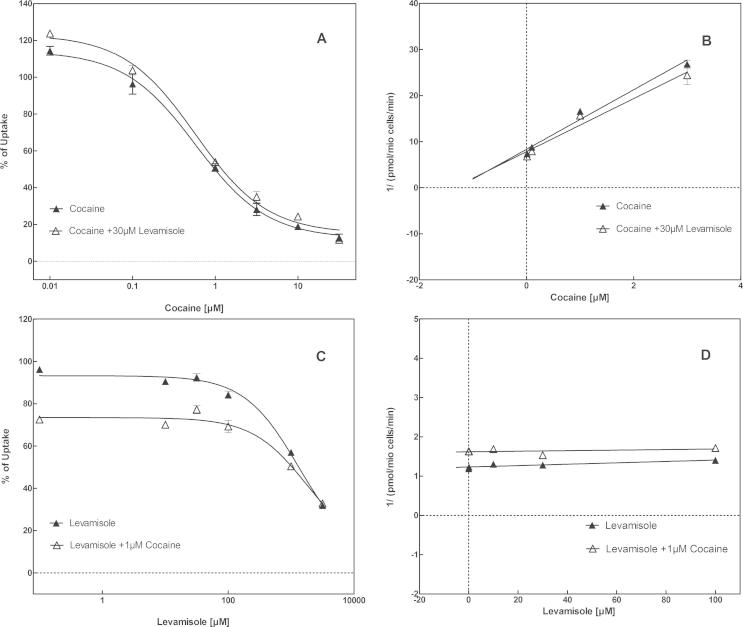
Determination of allosteric interaction between levamisole and cocaine by using uptake inhibition experiments. Uptake inhibitions with levamisole and cocaine were performed by using HEK 293 cells stably expressing human SERT. Uptake of substrate 5-HT at increasing cocaine concentrations at fixed levamisole concentrations (A) IC_50_ values for cocaine were 1.28 ± 1.17 μM in the absence and 1.71 ± 1.23 μM in the presence of 30 μM levamisole. Data in (A) were transformed into a Dixon-plot (B). Equally, uptake of 5-HT at increasing levamisole concentrations at fixed cocaine concentrations (C) is expressed as percentage of the maximum uptake without substance. IC_50_ values for levamisole were 129 ± 1.12 μM in the absence and 869 ± 1.47 μM in the presence of 1 μM cocaine. Data in (C) were transformed into a Dixon-plot (D).

**Supplementary Fig. 3.2:**
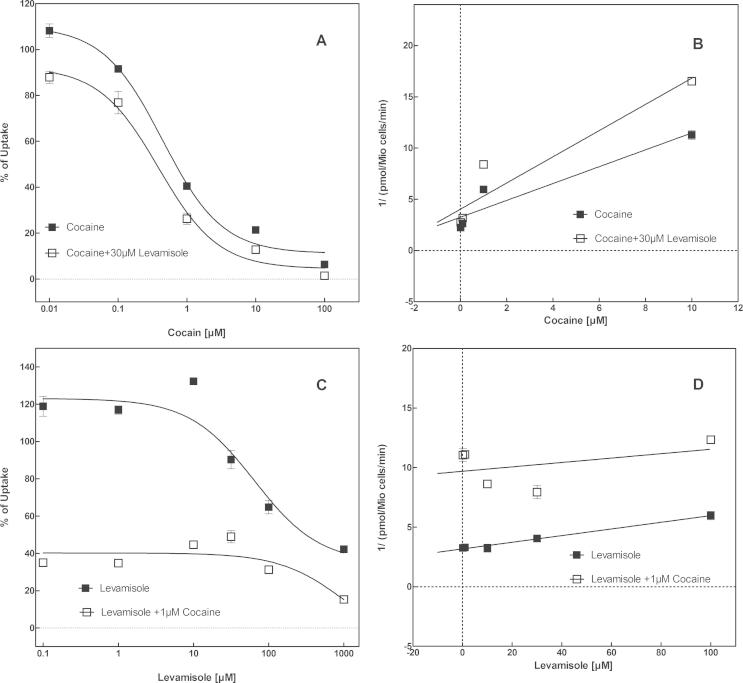
Determination of allosteric interaction between levamisole and cocaine by using uptake inhibition experiments. Uptake inhibitions with levamisole and cocaine were performed by using HEK 293 cells stably expressing human DAT. Uptake of substrate dopamine at increasing cocaine concentrations at fixed levamisole concentrations (A) IC50 values for cocaine were 0.84 ± 1.20 μM in the absence and 0.35 ± 1.17 μM in the presence of 30 μM levamisole. Data in (A) was transformed into a Dixon-plot (B). Equally, uptake of dopamine at increasing levamisole concentrations at fixed cocaine concentrations (C) is expressed as percentage of the maximum uptake without substance. IC50 values for levamisole were 476 ± 1.54 μM in the absence and 9.62 ± 1.98 μM in the presence of 1 μM cocaine. Data in (C) was transformed into a Dixon-plot (D).

**Fig. 3:**
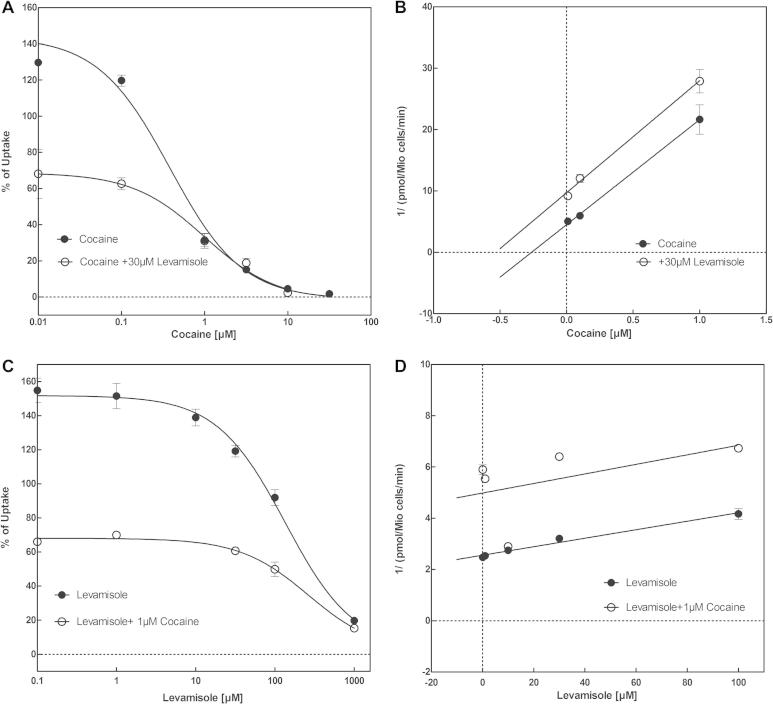
Determination of allosteric interaction between levamisole and cocaine by using uptake inhibition experiments. Uptake inhibitions with levamisole and cocaine were performed by using HEK 293 cells stably expressing human NET. Uptake of substrate MPP+ at increasing cocaine concentrations at fixed levamisole concentrations (A) is expressed as percentage of the maximum uptake without any inhibitory substance. IC_50_ values for cocaine were 0.80 ± 1.45 μM in the absence and 0.23 ± 1.47 μM in the presence of 30 μM levamisole. Data in (A) were transformed into a Dixon-plot (B) by expressing the reciprocal of MPP+ transported (pmol/million cells/min) as a function of cocaine at fixed concentrations of levamisole. Equally, uptake of MPP+ at increasing levamisole concentrations at fixed cocaine concentrations (C) is expressed as percentage of the maximum uptake without substance. IC_50_ values for levamisole was 521 ± 2.30 μM in the absence and 73 ± 1.47 μM in the presence of 1 μM cocaine. Data in (C) were transformed into a Dixon-plot (D) by expressing the reciprocal of MPP+ transported (pmol/million cells/min) as a function of levamisole at fixed concentrations of cocaine.

At the very least, the profile of levamisole is reminiscent of that the action of cocaine at the three transporters, with more prominent inhibition of DAT and NET, and somewhat lower affinity for SERT. Monoamine transporters have at least two binding sites, *i.e*., the SI-site, which corresponds to the substrate binding site proper, and the SII-site, which resides in the outer vestibule (Chen and Reith, 2004; Kristensen et al., 2011; Sarker et al., 2010). Accordingly, we explored the possibility that levamisole exerts an allosteric effect on the action of cocaine. We performed uptake-inhibition experiments in HEK293 cells expressing all three transporters and used increasing cocaine concentrations at a fixed levamisole concentration or *vice versa*. Representative experiments are shown in Fig. 3 for NET. The observations are consistent with binding of levamisole and cocaine to the same binding site. This can be best appreciated by examining the transformation of the data into Dixon plots (Segel, 1975). For this analysis the reciprocal of uptake velocity is plotted as a function of one inhibitor at a fixed concentration of the second inhibitor. Regardless of whether levamisole was varied at a fixed cocaine concentration (Fig. 3C and D) or – *vice versa* – cocaine was varied at a fixed levamisole concentration (Fig. 3A and B), the transformed data points fell onto parallel lines (Fig. 3B and D). This is indicative of mutually exclusive binding (Segel, 1975); intersecting lines ought to arise, if cocaine and levamisole can bind simultaneously, i.e., at two different sites. Identical experiments were performed for SERT and DAT (Supplementary Figs. S3.1 and S3.2) indicating as well mutually exclusive binding of levamisole and cocaine.

### 3.2 Is levamisole an inhibitor to NET, DAT and SERT only – or possibly a releaser?

**Fig. 4:**
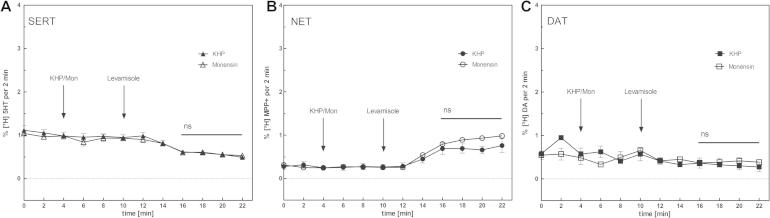
Releasing effect of levamisole. Substrate efflux after treatment with levamisole was measured for SERT (A), NET (B) and DAT (C). HEK 293 cells stably expressing human SERT, NET or DAT were preloaded with 0.4 μM [3H]5-HT, 0.1 μM [3H]MPP+, or 0.4 μM [3H]dopamine for 20 min at 37 °C in a final volume of 0.1 mL/well, transferred to small superfusion chambers (0.2 ml) and superfused with KRH buffer (25 °C, 0.7 ml × min^−1^). After a washout period of 40 min to reach stable baseline for efflux, 10 μM monensin or buffer (control) was added after 4 min followed by 100 μM levamisole (10 min). Collection of fractions was carried out in 2 min interval. At the end of the experiment, cells were lysed in 1% SDS. In the absence of monensin we could not detect substrate efflux for SERT (A) or DAT (C). However, we observed a slight increased substrate efflux for NET (B) in presence of levamisole compared to the control. Also, we could not detect an effect of monensine alone for any of the three transporters. Data are shown as mean ± SEM of three independent experiments. Two-way ANOVA (Bonferroni post-test) could not detect significant differences in substrate efflux between levamisole and control neither in NET (*p* = 0.945) nor SERT (*p* = 0.989) nor DAT (*p* = 0.678). IC_50_ values have been calculated using GraphPad Prism 5.04 non-linear regression.

Drugs that interact with neurotransmitter transporters can be either classified as cocaine-like inhibitors, which trap the transporter in the outward facing conformation and thus interrupt the transport cycle (Schicker et al., 2012), or amphetamine-like releasers. These raise extracellular monoamine concentrations by triggering substrate efflux (Sitte and Freissmuth, 2010). Levamisole is distantly related in structure to amphetamine. It is therefore conceivable that levamisole has a releasing action. We increased the sensitivity of our analysis by co-incubation of the cells with monensin (Baumann et al., 2013; Scholze et al., 2000; Sitte et al., 2000). Monensin is an ionophore that promotes electroneutral Na^+^/H^+^ exchange and therefore elevates intracellular Na^+^ in cells without altering the membrane potential. Since SERT, NET and DAT couple substrate transport with symport of Na^+^ and Cl^−^, elevation of intracellular Na^+^ accelerates substrate efflux (Sitte and Freissmuth, 2010). Applications of 5–20 μM monensin have been found to raise intracellular Na^+^ to 30–50 mM in HEK293 cells (Chen and Reith, 2004). In the absence of monensin, no efflux was observed in SERT (Fig. 4A) or DAT (Fig. 4C) expressing cells at a high levamisole concentration (100 μM); however, there was a slight increase in [^3^H]MPP^+^ in the superfusate collected from HEK293-NET cells (Fig. 4C). Importantly, the addition of monensin did not increase transporter-mediated efflux any further (Fig. 4). This argues for levamisole-mediated inhibition of reuptake of continuously released substrate rather than for a true releasing action. We previously observed similar spurious releasing effects with the selective serotonin reuptake inhibitor paroxetine on HEK293-cells expressing SERT (Scholze et al., 2000). To our knowledge, the experiments show for the first time that levamisole directly inhibits the human NET and to a lesser extent SERT and DAT. This inhibition is mediated by a low-affinity interaction with the same site, to which cocaine is bound and thus the SI site.

### 3.3 The levamisole-metabolite aminorex modulates NET, SERT and DAT in a different manner

**Fig. 5:**
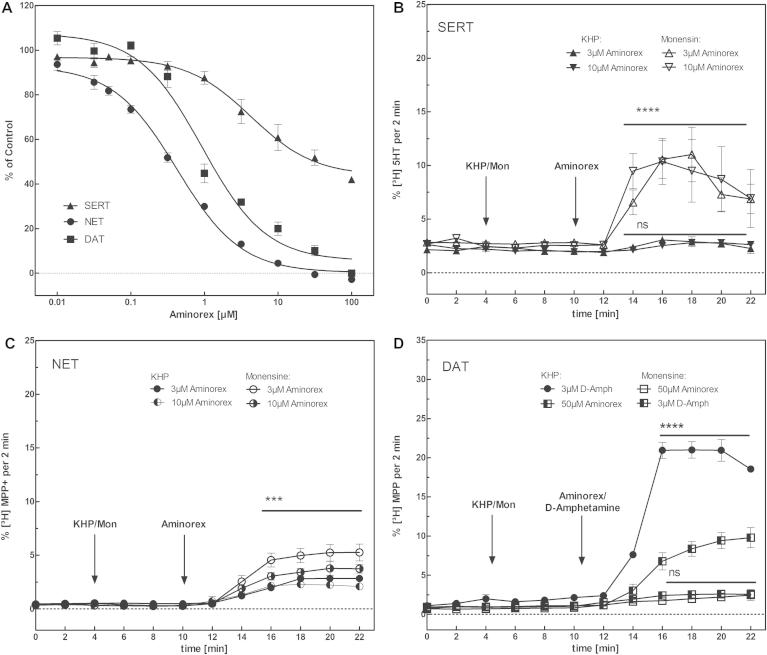
Inhibitory and releasing effect of aminorex. Uptake of substrate (SERT: 5-HT; NET: MPP+; DAT: dopamine) at increasing aminorex concentrations is shown in (A). IC_50_ values for aminorex (SERT: 18.39 ± 1.12 μM; NET: 0.33 ± 1.07 μM; DAT: 0.855 ± 1.20 μM) are suggesting a comparable inhibitory effect to cocaine for NET and DAT. For SERT the inhibitory effect was more than 10-fold higher compared to cocaine (all experiments were performed three times). Substrate efflux after treatment with aminorex was measured for SERT (B), NET (C) and DAT (D). In brief, HEK 293 cells stably expressing human SERT, NET or DAT were preloaded with 0,4 μM [3H]5-HT or 0,1 μM [3H]MPP+ for 20 min at 37 °C in a final volume of 0.1 mL/well, transferred to small superfusion chambers (0.2 ml) and superfused with KRH buffer (25 °C, 0.7 ml × min^−1^). After a washout period of 40 min to reach stable baseline for efflux, 10 μM monensin or buffer (control) was added after 4 min followed by 3 or 10 μM aminorex (SERT and NET) and 50 μM aminorex (DAT) respectively for 10 min. In addition we used 3 μM D-amphetamine as an positive control. Collection of fractions was carried out in 2 min interval. At the end of the experiment, cells were lysed in 1% SDS. While levamisole lead to an efflux of substrate via SERT and NET, which was enhanced by addition of monensin, no releasing effect of aminorex was observed at DAT (*p* = 0.9981). A slight increase could be observed in NET (*p* = 0.001) and aminorex could trigger a strong release in SERT (*p* < 0.0001). Data are shown as mean ± SEM of three independent experiments. *p*-values were calculated using a Two-way ANOVA (Bonferroni post-test).

**Fig. 6:**
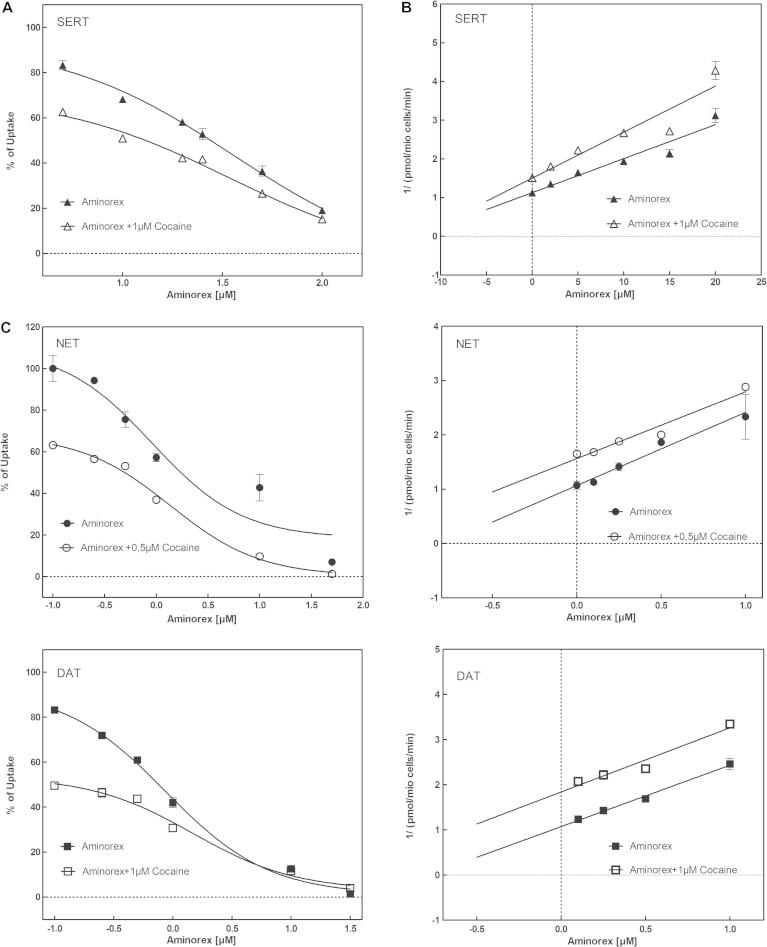
Determination of allosteric interaction between aminorex and cocaine by using uptake inhibition experiments. Uptake inhibitions with aminorex and cocaine were performed by using HEK cells stably expressing human NET. Uptake of substrate 5-HT, MPP+, and dopamine at increasing cocaine concentrations at fixed aminorex concentrations (A, C, and E) is expressed as percentage of the maximum uptake without substance. IC_50_ values for aminorex were 26.29 ± 1.03 μM for SERT (A), 1.97 ± 1.20 μM for NET (C), and 0.71 ± 1.05 μM for DAT (E) in the absence of cocaine. IC_50_ values in the presence of constant cocaine concentrations were as follows: SERT: 13.22 ± 1.08 μM at 1 μM Cocaine (A); NET: 0.406 ± 1.13 μM at 0.5 μM Cocaine (C); DAT: 0.20 ± 1.19 μM at 0.5 μM Cocaine. Data in (A, C, and E) were transformed into Dixon-plots (B, D, and F). The linear regression lines did not intersect, indicating no allosteric modulation of cocaine on aminorex binding. IC_50_ values have been calculated using GraphPad Prism 5.04 non-linear regression.

Administration of levamisole to race horses resulted in positive doping tests, because their urine contained aminorex (Barker, 2009). The metabolism of levamisole to the amphetamine-like compound aminorex was later confirmed to also occur in dogs and humans (Bertol et al., 2011; Hess et al., 2013). Hence, for the sake of comparison, we quantified the inhibition by aminorex of substrate uptake by NET, SERT or DAT (Fig. 5A). Interestingly, aminorex also preferentially blocked substrate uptake by NET (IC_50_: 0.33 ± 1.07 μM) and DAT (IC_50_: 0.85 ± 1.20 μM), while SERT was inhibited only at 20-fold higher concentrations (IC_50_: 18.39 ± 1.12 μM). Accordingly, the pattern of inhibition (NET > DAT >>> SERT) was reminiscent of the parent compound levamisole, but the inhibitory potency of aminorex was comparable to that of cocaine. To investigate if cocaine has an allosteric modulatory effect on aminorex, we performed uptake-inhibition experiments at increasing concentrations of aminorex in presence of fixed cocaine concentrations (Fig. 6). The resulting Dixon plots indicated that aminorex and cocaine bound in a mutually exclusive manner. In other words, there was not any appreciable allosteric modulatory effect in SERT, NET or DAT.

Aminorex is classified as an amphetamine-like substance, because it is chemically related to amphetamine and it suppresses feeding behavior in a manner similar to amphetamines. However, the neurochemical changes induced by aminorex differ from those of other appetite suppressants (Roszkowski and Kelley, 1963; Zheng et al., 1997). We therefore investigated its effects on substrate efflux by carrying out superfusion experiments in the presence and absence of monensin (10 μM). Interestingly, aminorex induced significant substrate release only in HEK293-SERT cells whereas efflux was completely absent in HEK293-DAT cells. HEK293-NET cells displayed only a slight response (Fig. 5B-D). Importantly, monensin enhanced efflux as predicted for an amphetamine-like releaser (Scholze et al., 2000). Taken together our experimental data showed that aminorex modulates the neurotransmitter transporters in different ways. On the one hand as an uptake inhibitor at NET and DAT with IC_50_ values of less than 1 μM but only weakly at SERT, on the other hand it elicits efflux in SERT without overtly affecting NET or DAT. The observation that aminorex causes significant substrate efflux only in SERT is coherent with the hypothesis that pulmonary hypertension, a major risk of aminorex consumption, is caused by dysregulation of peripheral serotonin transporters (Eddahibi and Adnot, 2002; Pollick, 1999) Hence, it may be assumed that aminorex has the potential to potentiate and/or prolong the effect of cocaine in its blocking propensity. Importantly, it may also prolong the cocaine sensations because it will elicit transporter-mediated substrate efflux owing to its amphetamine-like properties at times when cocaine is not present in the brain anymore (Jatlow, 1988; Moolchan et al., 2000). The pharmacokinetic parameters of levamisole are consistent with this hypothesis (Gwilt et al., 2000). This hypothesis is further supported by a recent analysis of human urine after levamisole administration, which showed that aminorex could be detected for up to 54 h (Hess et al., 2013).

Taken together, we demonstrate for the first time that levamisole directly inhibits the human NET. The metabolite aminorex itself modulates NET, DAT and SERT and results in a strong inhibition of NET and DAT substrate uptake and in substrate efflux at SERT. In addition we could not detect an allosteric modulatory effect of cocaine on aminorex.

### 3.4 Understanding the levamisole and aminorex–transporter interaction by a ligand-docking approach

**Fig. 7:**
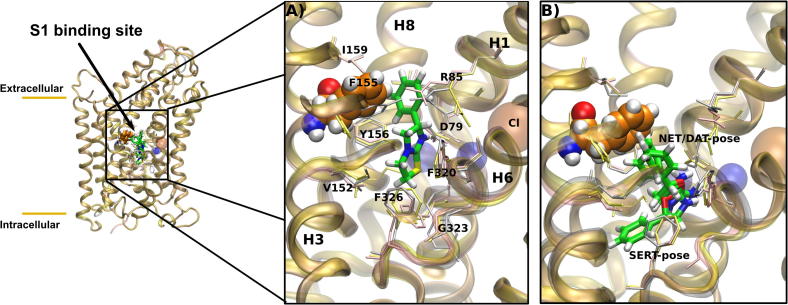
Docking poses of levamisole. Overlay of the docking poses of levamisole in NET (yellow), DAT (pink) and SERT (grey). The co-transported sodium (blue) and chloride (orange) ions are shown as spheres. Panel A and B show a zoom into the binding site. For clarity, only the docking poses in DAT is shown. The side chains that are within 5 Å from the ligand are shown as sticks Panel A: docking of levamisole into the substrate binding site (S1). The phenyl ring of levamisole directly contacts residue F151 in NET or residue Y155 in DAT. Binding of levamisole in SERT is lower compared to DAT and NET and differs by five residues between DAT and SERT (residues Y95, G100, I172, Y175 and T497 in SERT) and by four residues between NET and SERT (residue Y95, G100, I172 and T497 in SERT). Panel B shows overlaid docking poses of aminorex in the central binding site S1 of SERT, NET and DAT. Aminorex interacts with Y151 in NET and F155 in DAT. Docking pose in SERT showed that aromatic ring of aminorex is not orientated towards Y175, but inserted between I172 and Y95.

DAT, NET and SERT are very closely related (Beuming et al., 2006). The Dixon plots summarized in Fig. 3 provided conclusive evidence that cocaine and levamisole bound to the same site, namely SI, the substrate binding site proper. It is difficult to reconcile the high degree of conversation in the vicinity of the substrate binding site and the large differences in affinity of levamisole. Recently, we validated a ligand-based docking approach to probe the binding pocket of substrates in monoamine transporters (Seddik et al., 2013). Therefore, we used this computational approach to understand the discrimination by levamisole against SERT. The substrate binding sites of DAT and NET are almost identical. They differ only by one residue in helix 3, namely residue F151 in NET that corresponds to residue Y155 in DAT (Fig. 7A). Hence, we investigated, if the phenylalanine – tyrosine substitution explained the threefold difference in uptake inhibition. As levamisole has a p*K*_a_ of 7, we docked both the neutral and the protonated form of levamisole into the central substrate binding site of the neurotransmitter transporter. The positively charged amine functional group of serotonin, dopamine and norepinephrine has been found to interact with the sodium coordinating aspartate in the binding site. We made use of this interaction to reduce the search space for docking poses and imposed an interaction of the protonatable nitrogen of levamisole with the conserved aspartate residue (D75 in NET, D79 in DAT and D98 in SERT). Similar docking poses were observed for both protonation states of levamisole in all three transporters. The docking score showed the same ranking for both ligand protonation states. The highest affinity was predicted for NET (charged: −830 kcal/mol; neutral: −820 kcal/mol), followed by DAT (charged: −798 kcal/mol neutral: −792 kcal/mol) and SERT (charged: −697 kcal/mol neutral: −683 kcal/mol); nevertheless, scores alone have limited predictive power (Warren et al., 2006) and require confirmation by other means. This limitation, however, is less relevant in our approach, because the same ligand is docked into almost identical binding sites. The observed phenylalanine – tyrosine substitution between NET and DAT is very conservative, but it introduces a polar hydroxyl function by contrast with the hydrophobic phenylalanine side-chain. Importantly, the phenyl ring of levamisole directly contacts residue F151 in NET or residue Y155 in DAT in our docking poses, which is consistent with the experimental data. Our inhibition experiments showed that binding affinities of levamisole for SERT were lower when compared to that for NET and DAT. The binding site differs by five residues between DAT and SERT (residues Y95, G100, I172, Y175 and T497 in SERT) and by four residues between NET and SERT (residue Y95, G100, I172 and T497 in SERT). Levamisole was found to be in direct contact with four of these residues. We only observed that residue T497 was not in direct contact with the inhibitor. In line with our experimental findings, the difference in affinity between SERT and NET or DAT was therefore recapitulated by our computational approach.

The active metabolite of levamisole (aminorex) binds with comparable affinity to DAT and NET, while the affinity to SERT is lower (see Fig. 5). Aminorex is smaller than levamisole. During our docking studies of aminorex, we applied the same protocol as used for levamisole and identified docking poses in the central binding site S1. Both, neutral and positively charged forms of aminorex have been docked, as the p*K*_a_ of this psychostimulant is 7.4. We observed similar poses for both protonation states and discuss here the results of the positively charged state, as endogenous substrates are typically transported in their charged form. The positively charged nitrogen of aminorex interacts in a similar way with the aspartate (D75 in NET, D79 in DAT, D98 in SERT) as found for levamisole or nortriptyline in the recently published dDAT structure (Penmatsa et al., 2013).

The rank order of the binding energies scores (IFD score) compares favorably with the experimentally found affinities: NET (−822 kcal/mol), DAT (−789 kcal/mol) and SERT (−693 kcal/mol). Docking poses revealed overlapping geometries for the interaction of aminorex with NET and DAT (see Fig. 7B). Aminorex is in direct contact with Y151 in NET or F155 in DAT which could help to explain the observed differences in affinity. Importantly, the docking pose in SERT is different. We find that the aromatic ring of aminorex is not oriented towards the corresponding residue Y175, but inserted between I172 and Y95. Both residues differ in NET and DAT. We find in the corresponding positions V148 and F72 in NET and V152 and F76 in DAT. These docking results are in line with our experimental observation of the different behavior in the binding of aminorex to SERT compared to NET and DAT.

## 4 Conclusions

A large part of illicitly sold drugs are marketed in adulterated form; these commercialized preparations often may contain several additional, also pharmacologically active compounds. There are two obvious explanations why certain substances are used to adulterate illicit drugs: substances are added because they are cheap, have similar chemical appearance and taste and therefore increase the profit. Alternatively, the additives enhance the psychoactive effects of the drug by exerting a pharmacological effect *per se*. Accordingly, they contribute to the drug-specific reinforcement, gain more customers and thus increase profits. To our knowledge this work demonstrated for the first time that levamisole as cocaine adulterant itself directly inhibits the neurotransmitter transporters DAT, SERT and NET. Moreover, we found a cocaine-like effect of the levamisole metabolite aminorex at the DAT and the NET and an amphetamine-like effect at SERT. Therefore, it can be assumed that levamisole is used to prolong the effect of cocaine: it is possible that after the cocaine effect “fades out” the aminorex effect “kicks in”. However, the physiological consequences of combined cocaine-aminorex administration are still unclear. To our knowledge there are no reports on how the combination of cocaine and aminorex influences drug experience or brain physiology. It can be assumed that massive elevation of extracellular serotonin levels not only by inhibiting uptake (via cocaine) but also increasing efflux (via aminorex) can be the consequence.

The ‘checkit!’ program offers a glimpse into the epidemiology of the problem:

Two-thirds of the cocaine samples that were analyzed within the past year were contaminated with moderate to exceedingly high concentrations of levamisole. The latter highlight the risk inherent in adulteration of street drugs, namely the occurrence of severe or life-threatening intoxications. Therefore it is important to mention that consumption of cocaine adulterated with levamisole not only provokes severe agranulocytosis (Buchanan and Lavonas, 2012) but also induces the risk of pulmonary hypertension due to aminorex (Fishman, 1999b).

## Figures and Tables

**Fig. 1A:**
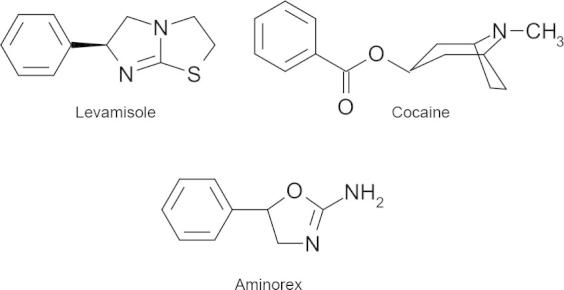
Chemical structures of cocaine, levamisole and aminorex.

**Fig. 1B:**
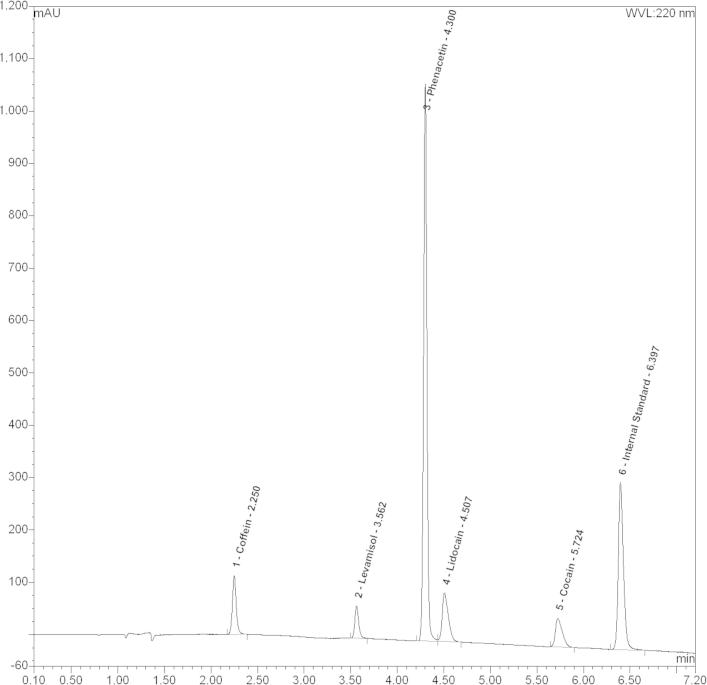
HPLC mass spectrometry. Representative HPLC chromatogram (UV detection trace at 220 nm) of a cocaine sample which was anonymously delivered to ‘checkit’. Besides cocaine it also contains significant amounts of caffeine, levamisole, phenacetin, and lidocaine.

**Table 1:** Prevalence of substances traced in 104 samples sold as cocaine.

Substance	Prevalence (%)
4-Methylethcathinone	1
Amphetamine	1
Benzocaine	2
Benzoylecgonine	51
*cis*-Cinnamoylcocaine	15
Caffeine	35
Hydroxyzine	1
Cocaine	93
Mephedrone	1
Levamisole	63
3,4-Methylenedioxy-*N*-methylamphetamine (MDMA)	2
3′,4′-Methylenedioxy-α-pyrrolidinobutyrophenone (MDPBP)	1
Lidocaine	18
Methylenedioxypyrovalerone (MDV)	2
Paracetamol	7
Phenacetin	43
Procaine	4
Tetracaine	1
*trans*-Cinnamoylcocaine	13
Unknown substance	14
